# Distribution of glomerular diseases in Taiwan: preliminary report of National Renal Biopsy Registry–publication on behalf of Taiwan Society of Nephrology

**DOI:** 10.1186/s12882-017-0810-4

**Published:** 2018-01-10

**Authors:** Hsien-Fu Chiu, Hung-chun Chen, Kuo-Cheng Lu, Kuo-Hsiung Shu, Taiwan Society of Nephrology

**Affiliations:** 1 Division of Nephrology, Department of Internal Medicine, Taichung Veterans General Hospital, Taichung, Taiwan; 2 Division of Nephrology, Department of Internal Medicine, Kaohsiung Medical University Hospital, Kaohsiung Medical University, Kaohsiung, Taiwan; 3 Faculty of Renal Care, College of Medicine, Kaohsiung Medical University, Kaohsiung, Taiwan; 4 Graduate Institute of Clinical Medicine, College of Medicine, Taipei Medical University, Taipei, Taiwan; 5 Division of Nephrology, Department of Internal Medicine, Cardinal Tien Hospital, School of Medicine, Fu-Jen Catholic University, New Taipei City, Taiwan; 6 Division of Nephrology, Department of Medicine, Tri-Service General Hospital, National Defense Medical Center, Taipei, Taiwan; 7 School of Medicine, Chung Shan Medical University, Taichung, Taiwan; 8 Division of Nephrology, Department of Internal Medicine, Lin Shin Hospital, No.36, Sec. 3, Hueijhong Rd., Nantun Dist, Taichung City, 40867 Taiwan, Republic of China

**Keywords:** Renal biopsy, Glomerulonephritis, IgA nephropathy

## Abstract

**Background:**

Despite the development of biomarkers and noninvasive imaging tools, biopsy remains the only method for correctly diagnosing patients with unexplained hematuria, proteinuria and renal failure. Renal biopsy has been performed for several decades in Taiwan; however, a national data registry is still lacking until 2013.

**Methods:**

The Renal Biopsy Registry Committee was established within the Taiwan Society of Nephrology in January 2013. A biopsy registry format, including basic demographic data, baseline clinical features, laboratory data, and clinical and pathological diagnosis was developed. Approval from the local institutional review board was obtained in each participating medical center.

**Results:**

From January 2014 to September 2016, 1445 renal biopsies were identified from 17 medical centers. 53.8% cases were reported in men. After excluding renal transplantation, renal biopsies were commonly performed in patients with primary glomerulonephritis (48.1%), secondary glomerulonephritis (36.2%), followed by tubulointerstitial diseases (12.3%) and vascular nephropathy (3.4%). Among primary glomerulonephritis, IgA nephropathy (26.0%), focal segmental glomerulosclerosis (21.6%), and membranous nephropathy (20.6%) were most frequently diagnosed. Diabetic nephropathy (22.4%) and lupus nephritis (21.8%) were the most common among secondary glomerulonephritis. Patients with minimal change disease and membranous nephropathy had heavier proteinuria than those with focal segmental glomerulosclerosis and IgA nephropathy (*P* < 0.001). Patients with minimal change disease had higher serum IgM and IgE levels. The most common cause of nephrotic syndrome in primary glomerular disease was membranous nephropathy (28.8%), followed by minimal change disease (28.2%). IgA nephropathy was the leading cause of chronic nephritic syndrome, acute nephritic syndrome, and persistent hematuria. The incidence of primary glomerulonephritis was approximately 2.19 in 100,000/year.

**Conclusions:**

This is the first report of the National Renal Biopsy Registry in Taiwan. IgA nephropathy is the most common primary glomerulonephritis, while membranous nephropathy is the most common cause of nephrotic syndrome. Primary glomerulonephritis distribution in Taiwan is slightly different from that in other Asian countries.

**Electronic supplementary material:**

The online version of this article (10.1186/s12882-017-0810-4) contains supplementary material, which is available to authorized users.

## Background

Based on the United States Renal Data System (USRDS) report, Taiwan has the highest incidence and prevalence of end-stage renal disease (ESRD) [1]. Diabetes mellitus (DM) (43.2%), chronic glomerulonephritis (GN) (25.1%), hypertension (8.3%), and chronic interstitial nephritis (2.8%) are the four major underlying diseases of ESRD in Taiwan [2]. DM and chronic GN are also the leading cause of ESRD in Japan and China [3, 4]. For chronic kidney disease, DM and hypertension are the most common etiologies in all developed and many developing countries, whereas chronic GN is more common in Asian countries, such as China, Japan, and Taiwan [5].

Accurate diagnosis for kidney disease is essential to decrease ESRD incidence and chronic kidney disease burden. Renal biopsy remains a powerful tool for correctly diagnosing patients with unexplained hematuria, proteinuria and renal failure. Renal biopsy has been performed for several decades in Taiwan; however, a national data registry is still lacking until 2013.

IgA nephropathy is the most frequent biopsy-proven glomerular disease in Japan, China, Singapore, Australia, the USA, and some European countries. In contrast, focal segmental glomerulosclerosis (FSGS) remains the most common biopsy-proven GN in Brazil and Saudi Arabia [6]. Owing to the improvement of public health and easier access to bacterial infection treatment, the frequency of membranoproliferative glomerulonephritis (MPGN) and post-infectious glomerulonephritis (PIGN) is decreasing in many countries [6–9]. To date, the distribution of glomerular diseases in Taiwan had not been documented based on renal biopsy.

Establishing a renal biopsy registry provides valuable epidemiological and clinical data of renal diseases with a histological diagnosis. It plays a vital role in research collaboration and preventive diseases programs, especially in the area of highest risk for DM, hypertension, and chronic GN**.** As a preliminary report of biopsy registry in Taiwan, we will demonstrate the clinicopathological correlations of biopsy-proven glomerular diseases, demographic data, laboratory data, and histopathological spectrum of the nephrotic syndrome. Primary glomerular disease may be induced by dysregulation of immune system, and previous study had reported that elevation of certain immunoglobulin levels actually correlated to specific GN and disease presentations [10–12]. Therefore, we also included immunologic profile of primary glomerular diseases in our registry database.

## Methods

In January 2013, the Renal Biopsy Registry Committee was established within the Taiwan Society of Nephrology. The committee consisted of 14 board members selected from **17** medical centers in Taiwan. Based on the report of the Taiwan Society of Nephrology, the number of renal biopsies in these 17 medical centers accounted for 79.7% of all renal biopsies in Taiwan in 2015. Approval from local institutional review board was obtained in each participating medical center. A biopsy registry format, including basic demographic data, baseline clinical features, laboratory data, clinical diagnosis and pathological diagnosis was developed.

Basic demographic data includes age, sex, and the presence of diabetes mellitus, hypertension, HBsAg and anti-HCV. Baseline clinical features were classified as follows: (1) acute nephritic syndrome, (2) rapidly progressive glomerulonephritis (RPGN), (3) persistent hematuria, (4) chronic nephritic syndrome, (5) nephrotic syndrome or unexplained proteinuria, (6) acute kidney injury, (7) drug-induced nephropathy, (8) kidney disease-associated vasculitis, (9) kidney disease associated with metabolic disorder, and (10) others. Acute nephritic syndrome was defined as hematuria, RBC casts, proteinuria (usually <3.5 g/day), mild degrees of reduced renal function, and hypertension. RPGN was defined as an acute nephritic syndrome with acute renal function decline within weeks to months. The chronic nephritic syndrome was defined as proteinuria (< 3.5 g/day) and hematuria persisting for more than 3 months. Nephrotic syndrome was defined as proteinuria more than 3.5 g/day with or without hypoalbuminemia and hyperlipidemia. Acute kidney injury was defined based on the Kidney Disease Improving Global Outcomes (KDIGO) guideline: increased in serum creatinine by ≧0.3 mg/dL within 48 h or increased serum creatinine to ≧1.5 times baseline 7 days prior or urine volume < 0.5 ml/kg/h for 6 h. Kidney disease associated vasculitis was defined as nephritic syndrome with the clinical features of systemic vasculitis, including palpable purpura, hemoptysis, rhinosinusitis, or neurologic symptoms associated with vasculitis.

Laboratory data and immunologic profile were collected at the time of biopsy, which included serum creatinine concentration, estimated glomerular filtration rate (eGFR) based on the Modification of Diet in Renal Disease (MDRD) equation, 24 h urine protein, hematuria, serum hemoglobin, and serum C3, C4, IgG, IgA, IgM, and IgE levels. Testing for antineutrophil cytoplasmic autoantibodies (ANCA) and anti-glomerular basement membrane was requested in specific cases.

Pathological diagnoses were classified into primary GN, secondary GN, tubulointerstitial disease, vascular nephropathy, others (sclerosing GN, inadequet biopsy, etc). Primary GN included (1) minimal change disease (MCD), (2) FSGS, (3) membranous glomerulonephritis (MGN), (4) IgA nephropathy, (5) mesangial proliferative glomerulonephritis (MsPGN) other than IgA nephropathy, (6) MPGN type 1,3 (7) MPGN type 2, (8) endocapillary proliferative glomerulonephritis (EnPGN), (9) crescentic glomerulonephritis (CreGN), type 1, (10) CreGN, type 2, and (11) CreGN, type 3. Type 1 CreGN is also known as anti-glomerular basement membrane (anti–GBM) disease or Goodpasture syndrome if combined with pulmonary hemorrhage. Type 2 CreGN is mediated by immune-complex. Type 3 CreGN, or pauci-immune RPGN is usually associated with ANCA vasculitis. Secondary GN consisted of lupus nephritis, diabetic nephropathy, amyloid nephropathy, thin basement membrane disease, Alport syndrome, and others. Tubulointerstitial diseases included acute tubular necrosis, acute tubulointerstitial nephritis, and chronic tubulointerstitial nephritis. Vascular nephropathy included thrombotic microangiopathy and hypertensive nephrosclerosis. Graft kidney biopsy was classified as renal transplantation.

The indications for renal biopsy varied among centers according to local practice. Biopsy samples were forwarded to pathologists in each medical center. They were examined by light, immunofluorescence and electron microscopy. One clinician in each renal unit was responsible for supplying data to the registry by mail.

Biopsy data of this preliminary report were obtained between January 5, 2014 and September 9, 2016.

### Statistical analysis

Continuous variables were represented using mean ± standard deviation or median (interquartile range, IQR). Categorical variables were shown as frequency (%). Continuous variables were examined for normality by using the Kolmogorov-Smirnov test. The χ^2^ test was used to compare qualitative variables. One-way analysis of variance (ANOVA) testing was performed to compare continuous variables normally distributed. The Kruskal-Wallis test was performed to compare continuous variables not normally distributed. All statistics were analyzed using SPSS software (version 21.0, Chicago, IL, USA). The *P*-values <0.05 were considered to be statistically significant. The annual incidence was defined as the total number of new cases per year related to the mean total population of the year, expressed as per million of population (p.m.p.).

## Results

### Demographic features

From January 2014 to September 2016, 1445 renal biopsies were identified from 17 medical centers in Taiwan. We identified 777 male samples, which accounted for 53.8% of the total samples. The mean age at biopsy was 48.4 ± 16.6 years. The median serum creatinine was 1.6 (IQR 0.9–3.3) mg/dL. The median daily urine protein was 2.7 (IQR 0.7–6.8) g/day, whereas 828 (57.3%) patients had hematuria.

### Clinical syndromes and pathological diagnosis

**Fig. 1:**
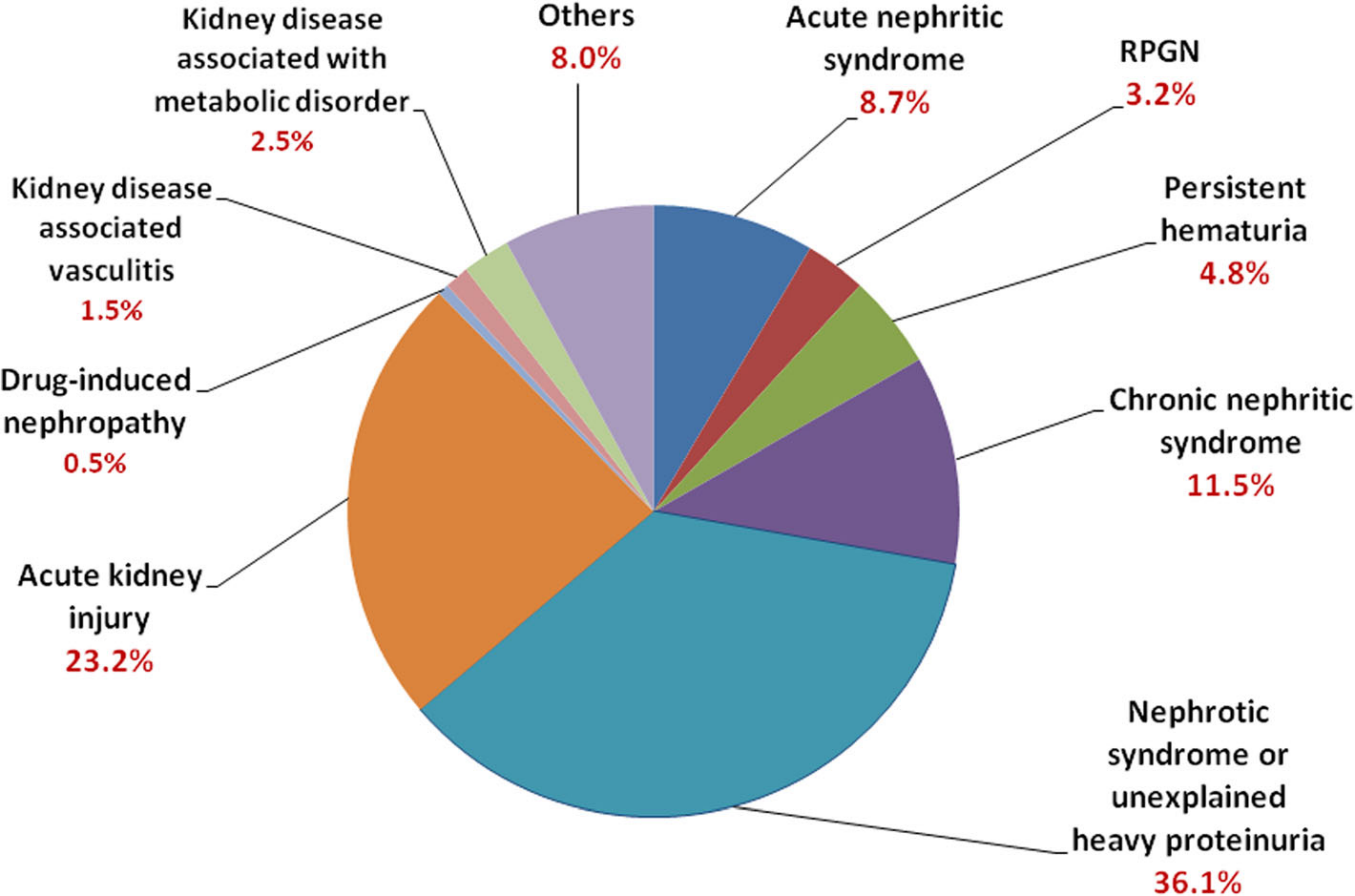
The frequency of classification of clinical diagnosis (*n* = 1467). Nephrotic syndrome, acute kidney injury, and chronic nephritic syndrome were the most common reasons for renal biopsy. RPGN, rapidly progressive glomerulonephritis syndrome

**Fig. 2:**
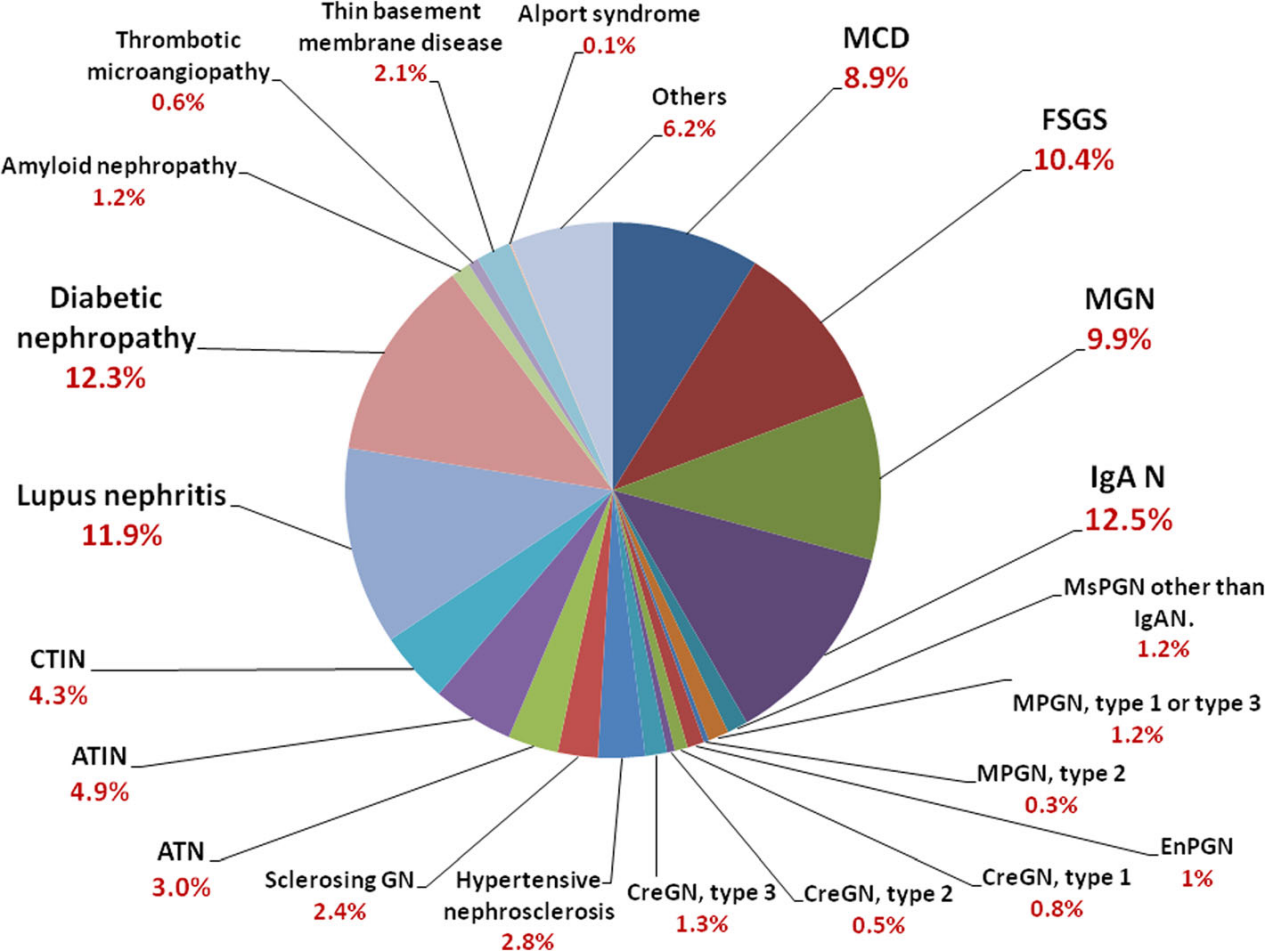
The frequency of different forms of biopsy-proven pathological diagnoses excluding renal transplantation (*n* = 1281). Primary glomerulonephritides accounted for 48.1%, secondary glomerulonephritides accounted for 36.2%, whereas tubulointerstitial diseases accounted for 12.3%. IgA nephropathy was the most frequent diagnoses among primary glomerulonephritides. On the other hand, diabetic nephropathy and lupus nephritis were the most common among secondary glomerulonephritides

**Fig. 3:**
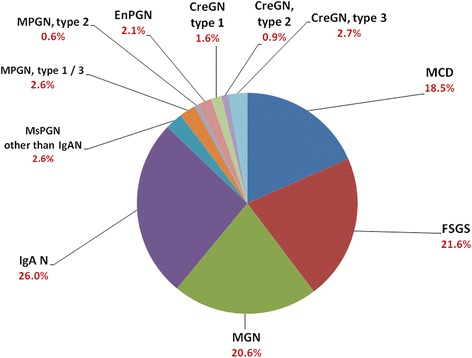
The frequency of biopsy proven primary glomerulonephritides (*n* = 616). IgA nephropathy, FSGS, MGN and MCD were the most common diagnoses

The most common clinical features at the time of biopsy were nephrotic syndrome or unexplained heavy proteinuria (36.1%), acute kidney injury (23.2%), chronic nephritic syndrome (11.5%), and acute nephritic syndrome (8.7%) (Fig. 1). Among patients who underwent renal biopsy in Taiwan, renal transplantation accounted for 221 cases **(**15.3%**).** After excluding renal transplantation, primary GN accounted for 48.1%, secondary GN accounted for 36.2%, tubulointerstitial diseases accounted for 12.3%, and vascular nephropathy accounted for 3.4%. (Fig. 2). Among primary GN, IgA nephropathy (26.0%), FSGS (21.6%), and MGN (20.6%) were the most frequent diagnoses (Fig. 3). On the other hand, diabetic nephropathy (22.4%) and lupus nephritis (21.8%) were the most common among secondary glomerulonephritis (Fig. 2).

### Clinicopathological correlations of primary glomerular diseases

As indicated in Table 1, the most common cause of nephrotic syndrome in primary glomerular disease was MGN (28.8%), followed by MCD (28.2%) and FSGS (24.8%). IgA nephropathy was the leading cause of chronic nephritic syndrome, acute nephritic syndrome, and persistent hematuria, accounting for 55.2%, 36.7%, and 76.7%, respectively. The most common etiology of RPGN of primary glomerular disease was crescentic glomerulonephritis (45.5%), followed by IgA nephropathy (15.2%), and FSGS (15.2%). FSGS (25.9%), IgA nephropathy (22.2%), and MCD (18.5%) were the most frequent primary glomerular disease presenting with acute kidney injury.

**Table 1:** The clinicopathological correlations of primary glomerular diseases (*n* = 599)

Histopathologic diagnosis	NS, *n* (%)	AKI, *n* (%)	CGN, *n* (%)	AGN, *n* (%)	PH, *n* (%)	RPGN, *n* (%)
IgA N	35 (10.7)	12 (22.2)	53 (55.2)	22 (36.7)	23 (76.7)	5 (16.1)
MGN	94 (28.8)	4 (7.4)	12 (12.5)	8 (13.3)	0 (0.0)	2 (6.5)
MCD	92 (28.2)	10 (18.5)	11 (11.4)	4 (6.7)	0 (0.0)	0 (0.0)
FSGS	81 (24.8)	14 (25.9)	11 (11.4)	8 (13.3)	5 (16.7)	2 (6.5)
MsPGN	4 (1.2)	2 (3.7)	5 (5.2)	2 (3.3)	1 (3.3)	0 (0.0)
CreGN	4 (1.2)	6 (11.1)	2 (2.1)	6 (10.0)	1 (3.3)	17 (54.8)
EnPGN	7 (2.1)	2 (3.7)	0 (0.0)	4 (6.7)	0 (0.0)	3 (9.7)
MPGN	9 (2.8)	4 (7.4)	2 (2.1)	6 (10.0)	0 (0.0)	2 (6.5)
Total	326 (100.0)	54 (100.0)	96 (100.0)	60 (100.0)	30 (100.0)	31 (100.0)

*NS*, nephrotic syndrome, *AKI* acute kidney injury, *CGN* chronic nephritis syndrome, *PH* persistent hematuria, *AGN* acute nephritis syndrome, *RPGN* rapidly progressive glomerulonephritis syndrome, *MCD* minimal change disease, *MGN* membranous nephropathy, *MsPGN* non-IgA mesangioproliferative glomerulonephritis, *MPGN* membranoproliferative glomerulonephritis, *CreGN* crescentic glomerulonephritis, *EnGN* endocapillary proliferative glomerulonephritis, *FSGS* focal segmental glomerulosclerosis

### Demographic data of primary glomerulonephritis

As shown in Table 2, the mean age at diagnosis for IgA nephropathy, MCD, FSGS, and MGN was 40.3 ± 14.4, 44.6 ± 18.8, 50.4 ± 17.1 and 58.1 ± 13.7 years, respectively (*P* < 0.001 for all pairwise). Among these four most common primary GN, male predominance was found. DM accounted for 11.4%~14.3%, HBsAg was positive in 4.4%~7.5% patients, and anti-HCV was positive in 0~3% patients. Hypertension accounted for 50.4% of patients with FSGS, which is more frequent than other primary GN (*P* = 0.01).

**Table 2:** Demographic data of the four major primary glomerulonephritides (*n* = 530)

	MCD (*n* = 114)	FSGS (*n* = 133)	MGN (*n* = 127)	IgAN (*n* = 156)	*P* value
Age, y	44.6 ± 18.8	50.4 ± 17.1	58.1 ± 13.7	40.3 ± 14.4	<0.001
Gender (male, %)	69 (60.5)	74 (55.6)	72 (56.7)	80 (51.3)	0.48
DM (n, %)	13 (11.4)	19 (14.3)	16 (12.6)	18 (11.5)	0.91
HTN (n, %)	38 (33.3)	67 (50.4)	49 (38.6)	58 (37.2)	0.01
HBsAg (n, %)	5 (4.4)	10 (7.5)	7 (5.6)	11 (7.1)	0.56
Anti-HCV (n, %)	2 (1.7)	4 (3.0)	5 (3.9)	0 (0.0)	0.11

Continues variable are represented by mean ± SD or median (interquartile range)

Categorical variables are shown as frequency (%)

### Laboratory data and immunologic profile of primary glomerulonephritis

As demonstrated in Table 3, the hemoglobin level in patients with MCD was higher than that in those with other primary GNs. Patients with FSGS and IgA nephropathy had higher serum creatinine than those with MCD and MGN (*P* < 0.001). Patients with MCD and MGN had heavier proteinuria compared with patients with IgA nephropathy and FSGS (*P* < 0.001). Microscopic hematuria was present in 72.4% patients with IgA nephropathy, which was more frequent compared with other primary glomerulonephritis (*P* = 0.02). Our registry also included immunologic profile, which were described below. The serum IgG level (mg/dL) in patients with MCD (median, 622.0; IQR, 337.0–899.7) and MGN (median, 632.0; IQR, 449.0–834.0) were lower than that in patients with FSGS (median, 925.5; IQR, 626.7–1235.0) and IgA nephropathy (median, 1126.0; IQR, 938–1366). Patients with IgA nephropathy had higher serum IgA levels (median, 369.0; IQR, 278.0–469.5 mg/dL), whereas patients with MCD had higher serum IgM (median, 121.0; IQR 82.4–173.5 mg/dL) levels. Serum IgE levels in patients with MCD was higher than that in those with MGN and IgA nephropathy (*P* = 0.04).

**Table 3:** Laboratory data and immunologic profile of the four major primary glomerulonephritides (*n* = 530)

	MCD (*n* = 114)	FSGS (*n* = 133)	MGN (*n* = 127)	IgAN (*n* = 156)	*P* value
Hemoglobin, g/dL	13.6 ± 2.3	12.4 ± 2.3	12.3 ± 2.3	12.5 ± 2.4	<0.001
C3, mg/dL	116.1 (96.1–138.0)	111.5 (90.9–133.7)	106.0 (94.0–126.0)	108.5 (92.4–125.6)	0.14
C4, mg/dL	28.1 (23.2–37.6)	28.6 (23.3–33.5)	27.9 (21.4–36.2)	25.6 (20.3–30.6)	0.03
IgG, mg/dL	622.0 (337.0–899.7)	925.5 (626.7–1235.0)	632.0 (449.0–834.0)	1126.0 (938–1366)	<0.001
IgA, mg/dL	260.4 (201.0–360.5)	288.5 (233.0–380.2)	267.0 (213.5–374.2)	369 (278.0–469.5)	<0.001
IgM, mg/dL	121.0 (82.4–173.5)	92.9 (65.4–130.0)	100.5 (64.1–132.5)	103.0 (73.4–140.0)	0.007
IgE, mg/dL	194.3 (30.5–573.0)	68.7.0 (23.3–245.5)	55.4 (23.2–207.0)	64.0 (19.3–173.0)	0.04
Cr, mg/dL	1.0 (0.7–1.6)	1.5 (0.9–2.5)	0.9 (0.7–1.7)	1.2 (0.8–2.1)	<0.001
eGFR, ml/min/1.73m^2^	78.1 (47.2–118.3)	63.7 (29.7–90.1)	81.3 (56.9–125.3)	59.4 (29.8–89.8)	0.03
Proteinuria, g/day	7.1 (2.5–12.5)	3.3 (1.5–7.6)	6.3 (4.0–10.7)	1.5 (0.6–2.9)	<0.001
Hematuria (*n*, %)	58 (50.8)	78 (58.6)	77 (60.6)	113 (72.4)	0.02

*eGFR* estimated glomerular filtration rate, calculated by MDRD equation. Continues variable are represented by mean ± SD or median (interquartile range). Categorical variables are shown as frequency (%)

### Histopathological spectrum of patients with nephrotic syndrome

MCD and lupus nephritis were the most frequent cause of nephrotic syndrome in the age groups of 14–24 and 25–44**,** respectively (Table 4). Among patients more than 45 years of age with nephrotic syndrome, the most frequent diagnosis was MGN, followed by FSGS. DM nephropathy accounted for 17.3% and 17.7% in patients between 45 and 59 years of age and patients more than 60 years of age, respectively. Renal amyloidosis was a rare diagnosis in patients with nephrotic syndrome, occurring exclusively in patients older than 45 years.

**Table 4:** The histopathological spectrum of patients with nephrotic syndrome at different ages (*n* = 531)

Pathological diagnosis	14–24 years *n* (%)	25–44 years *n* (%)	45–59 years *n* (%)	>60 years *n* (%)	Total *n* (%)	*P* value
MCD	22 (44.0)	31 (18.6)	20 (12.8)	18 (11.4)	91 (17.1)	<0.001
IgAN	7 (14.0)	15 (8.9)	10 (6.4)	2 (1.2)	34 (6.4)	0.003
FSGS	3 (6.0)	28 (16.7)	22 (14.1)	30 (18.9)	83 (15.6)	0.15
MGN	1 (2.0)	11 (6.6)	34 (21.8)	48 (30.4)	94 (17.7)	<0.001
LN	11 (22.0)	31 (18.6)	15 (9.6)	3 (1.9)	60 (11.3)	<0.001
MPGN	0 (0.0)	6 (3.6)	2 (1.3)	3 (1.9)	11 (2.1)	0.33
Renal amyloidosis	0 (0.0)	0 (0.0)	5 (3.2)	3 (1.9)	8 (1.5)	0.08
DM	0 (0.0)	21 (12.6)	27 (17.3)	28 (17.7)	76 (14.3)	0.01
Others	6 (12.0)	24 (14.4)	21 (13.4)	23 (14.6)	74 (13.9)	
Total	50 (100.0)	167 (100.0)	156 (100.0)	158 (100.0)	531 (100.0)	

*MCD* minimal change disease, *MGN* membranous nephropathy, *MPGN* membranoproliferative glomerulonephritis, *CreGN* crescentic glomerulonephritis, *FSGS* focal segmental glomerulosclerosis, *LN* lupus nephritis

### Annual incidence of glomerulonephritis

The population of Taiwan is around 23.5 million. This study involves children and we estimate that the biopsy population is around 80% of the whole population (18.8 million). The biopsy rate was around 43.9 biopsies per million population/year (pmp/yr). The incidence was 5.5, 4.7, 4.5, and 4.1 pmp/yr. for IgA nephropathy, FSGS, MGN, and MCD, respectively. The incidence of primary glomerulonephritis was 21.9 pmp/yr.

## Discussions

This preliminary report provides the epidemiological description and clinicopathological correlation for kidney disease diagnosed via renal biopsy in Taiwan over a period of 1.75 years (2014–2016) with 17 participating medical centers reporting 1445 renal biopsies. Based on the report of the Taiwan Society of Nephrology, renal biopsies in these 17 medical centers accounted for 79.7% of all renal biopsies in Taiwan in 2015. Therefore, this preliminary report was representative for analysis. The biopsy rate was 43.9 biopsy cases pmp/yr. in Taiwan. It is similar to that in Italy, Czech, Spain, and Cyprus, which is relatively high. [13, 14]

After excluding renal transplantation, renal biopsies were commonly performed in patients with primary GN (48.1%), secondary GN (36.2%), followed by tubulointerstitial diseases (12.3%) and vascular nephropathy (3.4%) (Fig. 2). Men are more represented in biopsy–proven renal disease (53.8%), which was similar in other countries such as UK (61%) [15], US (56.9%) [16], Italy (65%) [17], and China (63.3%) [7]. The present study also showed that IgA nephropathy (26.0%), FSGS (21.6%), and MGN (20.6%) were the most frequent diagnoses in primary GN. With the improvement of public health and adequate antibiotic treatment of pharyngeal infections, the incidence of MPGN and EnPGN decreased in many countries [6–9]. In Taiwan, the current report revealed that MPGN and EnPGN accounted for 3.2% and 2.1% only in primary GN, respectively.

There is a trend for increased prevalence of FSGS in many countries, such as Australia [18], India [19], Saudi Arabia [20], Thailand [21], Singapore [6], Japan [22] and the United States [16]. Ranking the second most common primary GN in Taiwan, FSGS accounted for 21.6% of primary GN, which is higher than that in other Asian countries such as Japan (9%) [8], China (3%) [7], Korea (8%) [9] and Singapore (15%) [6] but similar to US (20%) (Table 5). Among patients who presented with nephrotic syndrome, 15.6% were diagnosed with FSGS, which is again more frequent than that in China (6%) [7]. There is no definite explanation of this discrepancy, however, the increasing number of aging and obese population in Taiwan may partly account for the higher prevalence. Patients with FSGS had less proteinuria than those with MCD and MGN. Furthermore, hypertension was more common in FSGS than in other GN. We didn’t exclude adaptive FSGS from our biopsy database. Since adaptive FSGS is characterized by relative lower level of proteinuria, relative normal serum albumin level and obesity [23], adaptive FSGS may account for a substantial portion in our registry.

**Table 5:** International Comparison of Distribution of Primary glomerulonephritis

Country	Author	Year	Number of biopsies	IgA (%)	MCD (%)	FSGS (%)	MGN (%)	MsPGN (%)	EnPGN (%)	Others (%)
China	Zhou (7)	1993–2007	3331	54	11	3	15	11	–	6
Japan	Sugiyama (17)	2009–2010	4132	53	15	6	14	6	1	5
Korea	Chang (9)	1987–2006	1346	38	21	8	17	1	5	11
Singapore	Woo (6)	1998–2008	786	40	19	15	11	7	–	8
Thailand	Kanjanabuch (16)	2001–2004	506	31	–	25	13	–	–	31
Australia	Briganti (13)	1995–1997	1147	49	6	21	15	–	3	6
USA	Sundaraman (11)	1994–2003	195	25	5	20	10	12	8	20
Italy	Schena (12)	1987–1993	8287	35	8	12	21	–	–	25
India	Narasimhan (19)	1986–2002	3845	12	16	24	14	–	–	34
Saudi Arabia	Mitwalli (15)	1994–1999	127	10	9	35	4	25	16	2
Taiwan	Current study	2013–2015	616	26	18	22	21	3	2	9

*MCD* minimal change disease, *FSGS* focal segmental glomerulosclerosis, *MGN* membranous nephropathy, *MsPGN* non-IgA mesangioproliferative glomerulonephritis, *EnPGN* endocapillary proliferative glomerulonephritis

**Fig. 4:**
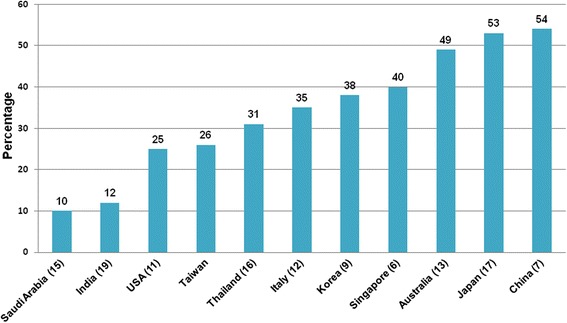
Comparison of frequency of IgA nephropathy in primary glomerular diseases in different countries. Adapted from Zhou et al. [7] with modification

IgA nephropathy is one of the most common primary GN worldwide. It is the most frequent biopsy-proven glomerular disease in Asia, including China (54%) [7], Japan (51%) [8], Korea (38%) [9], Singapore (40%) [6], Thailand (31%) [21] as well as in Taiwan. It is also the most common primary GN in many of the western countries such as Australia (49%) [18], the USA (25%) [16], Italy (35%) [17], France (37%) [24], Czech Republic (34%) and United Kingdom (39%) [6], but is relatively rare in Saudi Arabia [20], Brazil [25], and India [26], where FSGS remained the most common biopsy-proven GN [6] (Fig. 4, Table 5).

The high incidence of IgA nephropathy in many Asian countries may be explained as follows. First, Asians are more susceptible to IgA nephritis [27]. Geospatial differences in prevalence of genetic susceptibility loci was reported in global genome-wide association study. The frequency of the risk alleles for IgA nephropathy was highest in East Asians and lowest in African-Americans [28]. Second, the policies in screening kidney diseases and indication for renal biopsy vary among different countries. With loose criteria for biopsy, many early cases of IgA nephropathy presenting with minimal abnormalities in the urinalysis would be identified, thus raising the incidence of IgA nephropathy. In Taiwan, IgA nephropathy accounted for 6.4% of patients presenting with nephrotic syndrome (Table 4), which was higher than that of the Japan registry (3.8%~4.3%) [22, 29]. The mean eGFR at the time of biopsy was 63.9 mL/min, with a median proteinuria of 1.5 g /day (Table 3), which was also higher than that of the Japan Renal Biopsy Registry [22]. In summary, we speculated that IgA nephropathy was diagnosed at a relatively later stage in Taiwan.

Among all age groups, the most common cause of nephrotic syndrome in primary glomerular disease in Taiwan was MGN (28.8%), followed by MCD (28.2%) and FSGS (24.8%) (Table 4). This distribution is similar to results of other countries including China [7], Spain [30], and Italy [17], except for lower incidence of FSGS in China and Spain. In contrast, MCD is the leading cause of nephrotic syndrome in Korea [9] and Japan [22]. A possible explanation is that the mean age during biopsy in Korea was lower (36 years) than that in Taiwan (48.3 years), and that patients younger than 20 years comprised up to 12.1% in Japan, whereas they comprised only 3.5% in Taiwan. On the other hand, because MCD is known to predominantly distributed in younger patients with nephrotic syndrome, some patients with nephrotic syndrome may be prescribed corticosteroid before renal biopsy. Renal biopsy is reserved for those who do not have an adequate response to corticosteroids. As a consequence, the frequency of MCD may be underestimated in Taiwan.

MCD and LN were the leading causes of nephrotic syndrome in patients younger than 44 years. MCD is more frequently diagnosed in patients younger than 24 years (Table 4). Among patients older than 45 years, the most common primary GN that results in nephrotic syndrome was MGN, followed by FSGS, whereas the most frequent secondary GN was DM nephropathy. Renal amyloidosis occurred exclusively in older patients and usually presented with nephrotic syndrome. The mean age at diagnosis was 63.9 ± 11.7 years, and median urine protein was 8.2 g/day (IQR: 5.4–12.5).

Patients with IgA nephropathy had higher serum IgA levels than other primary GN, which had been documented before [10]. On the other hand, patients with MCD had higher serum IgM levels (*P* = 0.007) and relatively lower serum IgA levels (Table 3). A previous study demonstrated a similar result: serum IgG and IgA levels are significantly reduced in nephrotic patients with MCD, whereas serum IgM averaged more than twice the normal levels in patients with MCD [11]. The author concluded that the primary defect in MCD may be immunologic and could consist of deficiency in the T-cell function that mediates the conversion of IgM synthesis to IgG synthesis. Serum IgE levels in patients with MCD (mean: 639.2 mg/dL) was higher than those of other glomerulonephritides (*P* = 0.04). The result is consistent with our previous study [12], which indicated a significantly higher serum IgE level in MCD. Higher serum IgE level was usually associated with more frequent relapse or steroid resistance [12]**.** A lower overall inhibition rate on IgE synthesis, which is related to a significantly lower activity of IgE–specific suppressor factors may play a role [31]. These findings suggest there is a T cell disorder in some of the primary GN with high serum IgE, especially in MCD.

RPGN accounted for 3.2% of all the clinical syndromes in this preliminary report. CreGN comprised 54.8% of patients with RPGN. Anti-glomerular basement membrane (anti–GBM) disease or Goodpasture syndrome if combined with pulmonary hemorrhage, comprised 27.3%, whereas immune complex mediated CreGN, comprised 18.2%. Pauci-immune RPGN, which was usually associated with ANCA vasculitis, was reported 54.5%. Among pauci-immune RPGN, (total case number = 17 in this preliminary report), 11 cases (64.7%) were ANCA-MPO positive, 2 cases (11.7%) were ANCA-PR3 positive. 2 patients (64.7%) were “dual positive” for ANCA-PR3 and ANCA-MPO, and 2 patients (11.7%) were ANCA negative. The mean age at diagnosis was 58.5 ± 14.2 years (median 60.4, IQR 51.2–69.9); the mean serum creatinine was 5.6 ± 4.3 mg/dL (median 4.76, IQR 2.4–8.5); the median daily urine protein was 2.8 g (IQR 1.5–5.6). A previous study revealed that granulomatosis with polyangitis (GPA) is less common than microscopic polyangitis (MPA) in non–Europeans [32]. GPA is not as common as MPA in Japan and China [33], and this preliminary report demonstrated a similar epidemiological pattern in Taiwan.

The incidence was 5.5, 4.7,4.5, and 4.1 pmp/yr. for IgA nephropathy, FSGS, MGN, and MCD, respectively. The incidence of primary glomerulonephritis was 21.9 pmp/yr. Compared with the incidence of primary GN worldwide, McGrogan et al. demonstrated a mean incidence of 2.5, 0.8, 1.2, and 0.6 /100,000/year for IgA nephropathy, FSGS, MGN, and MCD, respectively [34].

The strength of this study is that it recruited biopsies from 17 medical centers, which accounted for 79.7% of all renal biopsies in 2015, thus should be representative for epidemiological analysis for Taiwan, and this is the first report of the national data. However, the current study has several limitations. Our statistics may underestimate the true incidence of GN for two reasons. First, we do not have a population screening program to detect silent cases of GN, which is practiced in Japan. Diseases with asymptomatic urinary abnormality or microscopic hematuria such as IgA, MGN, and FSGS may be diagnosed at a later stage. Secondly, biopsy policies may vary from center to center, and renal biopsy is often not performed when the therapeutic advantage is judged as low. For example, patients with steroid– sensitive proteinuria in children, hematuria without proteinuria, PIGN, and diabetic nephropathy would not undergo renal biopsy in some of the medical centers. Therefore, the incidence of MCD, IgA nephropathy, PIGN, and diabetic nephropathy may be underestimated. Actually, there are several factors influencing the variability of biopsy practice worldwide, including heterogenecity of indication of renal biopsy, social and economic status, lack of renal biopsy data collection, which was just reviewed by Fiorentino et al. [13].

## Conclusions

In conclusion, the preliminary report from the renal biopsy registry provides valuable epidemiological and clinical data on renal diseases with a histological diagnosis in Taiwan. Primary GN was the leading cause of renal biopsy, and IgA nephropathy, FSGS, MGN were the most common diagnoses. The most frequent etiology of nephrotic syndrome of all ages was MGN, followed by MCD and FSGS. MGN and renal amyloidosis should be considered in older patients with nephrotic syndrome. Positive ANCA-MPO was more frequent than positive ANCA-PR3 in the CreGN subgroup. The relatively higher disease activity at presentation in IgA nephropathy probably implies a late diagnosis. For early diagnosis, further studies focusing on the timing and policy of kidney biopsy may be warranted.

## Additional file

Additional file 1: Institutional review boards (IRB) that approved this study and approval codes. (DOC 31 kb)

## Abbreviations

ANCA: Antineutrophil cytoplasmic autoantibodies
CreGN: Crescentic glomerulonephritis
DM: Diabetes mellitus
eGFR: Estimated glomerular filtration rate
EnPGN: Endocapillary proliferative glomerulonephritis
ESRD: End-stage renal disease
FSGS: Focal segmental glomerulosclerosis
GN: Glomerulonephritis
MCD: Minimal change disease
MGN: Membranous glomerulonephritis
MPGN: Membranoproliferative glomerulonephritis
MsPGN: Mesangial proliferative glomerulonephritis
PIGN: Post-infectious glomerulonephritis
USRDS: United States Renal Data System
