# Supplementary material for: Distribution of glomerular diseases in Taiwan: preliminary report of National Renal Biopsy Registry–publication on behalf of Taiwan Society of Nephrology
Source: BMC Nephrol. 2018 Jan 10;19:6. doi: 10.1186/s12882-017-0810-4 (PMC5764016; doi:10.1186/s12882-017-0810-4)
Supplement: Additional file 1: — Institutional review boards (IRB) that approved this study and approval codes. (DOC 31 kb) [file 12882_2017_810_MOESM1_ESM.doc]

**Institutional review boards (IRB) that approved this study and approval codes**

Taichung Veterans General Hospital, IRB No: CE15125B

Changhua Christian Hospital, IRB No: 150209

China Medical University Hospital, IRB No: CMUH104REC1-001

Kaohsiung Veterans General Hospital, IRB No: 15-CT4-03(150128-1)

Kaohsiung Medical University Hospital, IRB No: KMUHIRB-G(1)-20160017

Tri-Service General Hospital, IRB No: 2-104-05-078

National Taiwan University Hospital, IRB No: 201601077RINA

Kaohsiung Chang Gung Memorial Hospital, IRB No: 104-3124B

Chiayi Chang Gung Memorial Hospital, IRB No: 104-3124B

Linkou Chang Gung Memorial Hospital, IRB No: 104-3124B

National Cheng Kung University Hospital, IRB No: B-ER-104-048

Taoyuan General Hospital, Ministry of Health and Welfare, IRB No: TYGH104004

Taipei Veterans General Hospital, IRB No: 2015-05-003AC

Chung Shan Medical University Hospital, IRB No: CS12035

Kuang Tien General Hospital, IRB No: KTGH10645

Chi Mei Hospital, IRB No: 11522
